# Long-Term Outcomes of Orthotopic Neobladder Versus Ileal Conduit Urinary Diversion in Robot-Assisted Radical Cystectomy (RARC): Multicenter Results from the Asian RARC Consortium

**DOI:** 10.1245/s10434-024-15396-5

**Published:** 2024-05-27

**Authors:** Chris Ho-ming Wong, Ivan Ching-ho Ko, Seok Ho Kang, Kousuke Kitamura, Shigeo Horie, Satoru Muto, Chikara Ohyama, Shingo Hatakeyama, Manish Patel, Cheung-Kuang Yang, Kittinut Kijvikai, Lee Ji Youl, Hai-ge Chen, Rui-yun Zhang, Tian-xin Lin, Lui Shiong Lee, Jeremy Yuen-chun Teoh, Eddie Chan

**Affiliations:** 1 S.H. Ho Urology Centre, Department of Surgery, Faculty of Medicine, Clinical Sciences Building, Prince of Wales Hospital, New Territories, The Chinese University of Hong Kong, Hong Kong SAR, China; 2 Department of Urology, Korea University Anam Hospital, Seoul, Republic of Korea; 3 Department of Urology, Juntendo University Graduate School of Medicine, Tokyo, Japan; 4 Department of Urology, Hirosaki University, Hirosaki, Japan; 5 Department of Urology, The University of Sydney, Sydney, Australia; 6 Department of Urology, Taichung Veterans General Hospital, Taichung, Taiwan; 7 Department of Urology, Ramathibodi Hospital, Mahidol University, Salaya, Thailand; 8 Department of Urology, Catholic University of Korea, Seoul, Republic of Korea; 9 Department of Urology, Renji Hospital, School of Medicine, Shanghai Jiaotong University, Shanghai, China; 10 Department of Urology, Sun Yat-Sen Memorial Hospital, Sun Yat-Sen University, Guangzhou, China; 11 Department of Urology, Sengkang General Hospital, Singapore, Singapore

**Keywords:** Robot assisted radical cystectomy, Ileal conduit, Orthotopic neobladder, Non-metastatic bladder cancer

## Abstract

**Purpose:**

Robot-assisted radical cystectomy (RARC) has gained traction in the management of muscle invasive bladder cancer. Urinary diversion for RARC was achieved with orthotopic neobladder and ileal conduit. Evidence on the optimal method of urinary diversion was limited. Long-term outcomes were not reported before. This study was designed to compare the perioperative and oncological outcomes of ileal conduit versus orthotopic neobladder cases of nonmetastatic bladder cancer treated with RARC.

**Patients and Methods:**

The Asian RARC consortium was a multicenter registry involving nine Asian centers. Consecutive patients receiving RARC were included. Cases were divided into the ileal conduit and neobladder groups. Background characteristics, operative details, perioperative outcomes, recurrence information, and survival outcomes were reviewed and compared. Primary outcomes include disease-free and overall survival. Secondary outcomes were perioperative results. Multivariate regression analyses were performed.

**Results:**

From 2007 to 2020, 521 patients who underwent radical cystectomy were analyzed. Overall, 314 (60.3%) had ileal conduit and 207 (39.7%) had neobladder. The use of neobladder was found to be protective in terms of disease-free survival [Hazard ratio (HR) = 0.870, *p* = 0.037] and overall survival (HR = 0.670, *p* = 0.044) compared with ileal conduit. The difference became statistically nonsignificant after being adjusted in multivariate cox-regression analysis. Moreover, neobladder reconstruction was not associated with increased blood loss, nor additional risk of major complications.

**Conclusions:**

Orthotopic neobladder urinary diversion is not inferior to ileal conduit in terms of perioperative safety profile and long-term oncological outcomes. Further prospective studies are warranted for further investigation.

The most common approaches of urinary diversion adopted in RARC are ileal conduit and orthotopic neobladder. Ileal conduit had been widely practiced with safety and oncological efficiency taken into consideration^11^ Contrastingly, orthotopic bladder typically appeals to some patients because of its ability to maximally retain continence function, with benefits of better body image and enhanced quality of life in a carefully selected population.^12^ However, comparative literature on perioperative or long-term oncological outcomes are heavily lacking. Most studies focused on the quality-of-life domains^13,14^ only.

The authors were concerned about whether additional evidence on the implications of urinary diversion methods on long-term oncological implications can be harvested. The use of orthotopic neobladder following RARC had been suggested to associate with inferior oncological outcomes. This was postulated to be the consequence of longer time under pneumoperitoneum, more complex maneuvers, and manipulations of the specimen, and hence a greater likelihood of tumor seeding within the peritoneal space. In the short term, perioperative outcomes of neobladder were also scrutinized. While neobladder appeared to be a more challenging procedure, especially when coupled with intracorporeal urinary diversion that became more commonly done in the era of robotic cystectomy,^15^ it was frequently criticized as being associated with increased blood loss and inferior safety profiles in some studies.^16^ This often brought about hesitation in choosing neobladder as the method of urinary diversion in RARC.

The Asian Robotic Assisted Radical cystectomy (RARC) consortium was a registry formed in 2017 by a number of Asian expert centers to look into the potential benefits and morbidities associated with RARC. Up to date there were more than 500 cases recorded in the registry. In the following analysis, we aimed to investigate the safety profile and oncological outcomes of neobladder compared with ileal conduit to truly dissect the merits and drawbacks of either procedure. We would like to investigate and compare the perioperative and long-term survival outcomes of neobladder versus ileal conduit following RARC for nonmetastatic bladder cancer.

## Patients and Methods

There were nine centers that participated in the Asian RARC registry. Consecutive nonmetastatic bladder cancer patients who were treated with RARC from the year 2007 to 2020 were analyzed. All cases within the registry with radical cystectomy performed for nonmetastatic diseases were retrieved for analysis. Cases that were managed with open radical cystectomy or laparoscopic radical cystectomy were excluded. Procedures that did not utilize ileal conduit or neobladder for urinary diversion were also excluded. A standardized electronic form had been established to record patient background, disease characteristics, operative details, perioperative outcomes, oncological outcomes, and survival data. Disease characteristics included disease staging and results of systemic workup. The reporting of T (tumor) and N (nodal) stages in our analysis was based on specimen review (pathological staging). In cases where lymph node dissection was not performed during radical cystectomy, N stage was reported as clinical staging from preoperative imaging investigation. Patient characteristics included age, comorbidities, and American Society of Anesthesiologists (ASA) status. Operative details recorded were the surgical approach, method of urinary diversion, console time, estimated blood loss, and intraoperative adverse events (if any). Postoperative details included time of diet resumption, day of discharge, instances of complications, and the need of 30-day readmission. Postoperative complications were defined as early postoperative events (within 30 days). They were graded according to the Clavien–Dindo classification system. Reporting of complications was based on clinical examination documented by physicians and the review of patient charts. Documentation, grading, and reporting of the complications from our cohort fulfilled the European Association of Urology (EAU) quality criteria for accurate and comprehensive reporting of surgical outcomes.^17^

The cohort was divided into the (1) ileal conduit group and the (2) orthotopic neobladder group. While the registry included cases from multiple centers, there were no specified criteria to guide the use of either of the urinary diversion methods. Urinary diversion was performed according to the preference and decision of each participating center. The steps of the surgical procedures and the techniques utilized were also based on individual center preference. Primary outcomes included disease-free survival and overall survival. Disease‐free survival was defined as the time from robotic cystectomy until first recurrence, progression of disease, metastasis, or death, whichever happened first. Oncological outcomes and survival data were collected during each follow-up. Death was defined as all-cause mortality. Patients that were alive without evidence of disease were censored on the date of last follow-up. Secondary outcomes included postoperative complications, blood loss, length of stay, and rates of readmission.

Statistical analyses were performed with SPSS version 24.0 (IBM). The recommendations for conducting data analysis were applied.^18^ Categorical variables were reported with descriptive statistics and percentages. Continuous variables were reported with median with interquartile ranges or mean with standard deviation and standard error of means. The Chi-square test and Fisher’s exact test were used to examine statistical significances. A *p*-value of 0.05 is considered statistically significant. Multivariate regression analysis was performed to identify any confounding factors for outcomes. Two sets of multivariate regression analysis models were adopted to assess the effect of urinary diversion method on (1) disease free survival (DFS) and (2) overall survival (OS). Covariates included pathological T stage, pathological N stage, tumor histology (according to the WHO 2004 criteria),^19^ use of neoadjuvant chemotherapy, need of adjuvant therapy post operation, preoperative hydronephrosis (unilateral or bilateral), preoperative renal impairment (defined by estimated glomerular filtration rate < 60 mL/min/1.73 m^2^), presence of diabetes at the time of operation, smoking history, ASA status, and patient age at the time of operation. Factors that demonstrated statistical significance in univariate analysis were fitted to the multivariate regression model. The number of covariates met the criteria for the model preventing overfitting in the performed multivariate regression analyses.^20^

## Results

From the year 2007 to 2020, there were 521 patients with RARC performed in nine centers in Asia that were managed with ileal conduit or neobladder. Out of the 521 cases, 314 patients (60.3%) had ileal conduit and 207 patients (39.7%) received orthotopic neobladder urinary diversion.

Patient characteristics were comparable between the two groups. The proportions of diabetes and smoking history were similar. The neobladder group had a relatively younger mean age compared with the ileal conduit group (61.6 versus 69.2 years, *p* < 0.001). The proportions of ASA status of 3 or above for patients in the neobladder group was less than the conduit group (5.9% versus 18.6%, *p* < 0.001). Disease characterizers are similar, including the portion of preoperative hydronephrosis. The neobladder group had more patients receiving neoadjuvant chemotherapy (6.2% versus 25.0%, *p* = 0.006). The pathological T and N stages were similar between the two groups. Around one-third of the patients had T3 or above disease in either of the groups. More than 80% of the cases had high grade histology in the specimen review. The details are listed in Table 1.

**Table 1:** Patient, disease, and operative characteristics

	Ileal conduit		Neobladder		*P*-value
	*N*	%/SD	*N*	%/SD	
Number of patients, %	314	60.3%	207	39.7%	
Mean age, SD	69.2	9	61.6	9.2	**< 0.001**
Sex (male:female)	262:52		193:14		**0.001**
Mean BMI (m^2^/kg), SD	24.6	3.5	24.5	3.1	0.455
Smoking history, %	150	48.4%	116	57.7%	**0.039**
Diabetes mellitus, %	78	26.1%	48	23.8%	0.173
Preoperative renal impairment, %	45	15.6%	14	9.8%	**0.006**
Preoperative hydronephrosis, %	51	16.3%	26	12.6%	0.5
History of abdominal surgery, %	49	17.4%	24	11.6%	**0.1**
History of pelvic radiotherapy, %	12	4.2%	4	2.1%	0.208
ASA, %					**< 0.001**
1	41	13.9%	60	29.4%	
2	200	67.6%	132	64.7%	
3 +	55	18.6%	12	5.9%	
Neoadjuvant chemotherapy, %	78	25.0%	75	36.2%	**0.006**
Preoperative radiotherapy, %	12	3.8%	2	1.0%	0.09
Lymph node dissection performed, %	311	99.0%	206	99.5%	0.546
Console time (mean), SD	336	113.0	388	118.0	**< 0.001**
High grade histology, %	227	84.1%	133	82.6%	0.692
pT stage, %					**0.002**
T0	21	6.7%	6	2.9%	
Ta/is	56	18.0%	67	32.4%	
1	50	16.0%	37	17.9%	
2	65	20.8%	41	19.8%	
3	88	28.2%	56	27.1%	
4	32	10.3%	10	4.8%	
pN+, %	19	7.3%	6	3.4%	**0.011**
Mean number of retrieved nodes, SEM	19.7	0.7	42.4	1.0	**< 0.001**

Significant values are given in bold (*P*-value ≤ 0.05)

*SD* standard deviation, *BMI* body mass index, *ASA* American Society of Anesthesiologists classification, *WHO* World Health Organization classification of bladder cancer, *pT stage* pathology tumor staging, *pN+* pathological nodal positive, *SEM* standard error of means.

Table 2 described the early postoperative outcomes of our cohort. The mean operative blood loss was almost identical between ileal conduit and neobladder (482 versus 484mL, *p* = 0.958). The ileal conduit group was associated with a shorter length of stay (14.2 versus 19.9 days, *p* < 0.001). Contrastingly, the neobladder group was associated with a lower rate of 30-day readmission (14.4% versus 22.6%, *p* = 0.093), although statistically insignificant. The rates of Clavien–Dindo 3 or 4 complications were similar at 16.8 versus 18.0% (*p* = 0.072).

**Table 2:** Early postoperative outcomes

	Ileal conduit		Neobladder		*P*-value
	*N*	%/SD/SEM	*N*	%/SD/SEM	
Mean intraoperative blood loss (mL), SD	482	425	484	383.0	0.958
Mean length of stay (days), SEM	14.2	0.637	19.9	0.892	**< 0.001**
Mean time to solid food mean (days), SEM	5.1	0.29	6.1	0.419	0.06
30-day readmission, %	67	22.6%	29	14.4%	0.093
Complications, %					0.072
No complications reported	150	52.4%	82	42.3%	
Clavien–Dindo grade 1/2	88	30.8%	77	39.7%	
Clavien–Dindo grade 3/4	48	16.8%	35	18.0%	

Significant values are given in bold (*P*-value ≤ 0.05)

*SD* standard deviation, *SEM* standard error of means

**Fig. 1:**
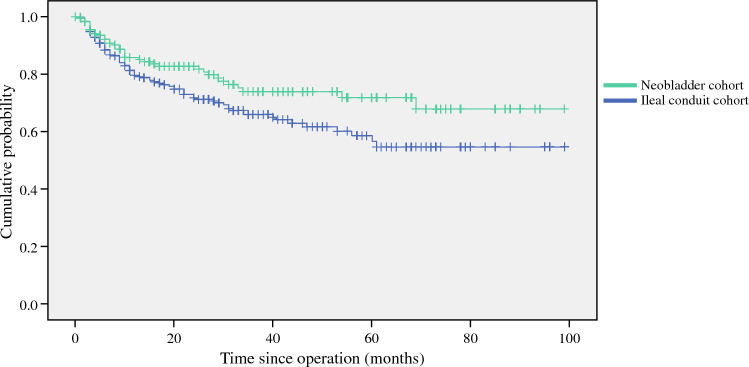
Kaplan–Meier survival curves of disease-free survival of the ileal conduit and neobladder cohorts. Hazard ratio = 0.661; 95% CI = 0.451–0.969; *p*-value = 0.032. *CI* confidence interval

We attempted to analyze whether different methods of urinary diversion were related to differences in survival outcomes. In our analysis on disease-free-survival, we observed a superior outcome in the neobladder group compared with the ileal conduit group [hazard ratio = 0.661, 95% confidence interval (CI) = 0.451–0.969, *p* = 0.032]. Results were represented in the form of a Kaplan–Meier survival analysis (Fig. 1). Further analysis was done to evaluate the factors related to the differential disease-free survival in these two groups of patients. At univariate analysis, it was found that use of ileal conduit (compared with neobladder), higher tumor T stage, pathological nodal stage, high grade histology, presence of preoperative hydronephrosis, and use adjuvant therapy were associated with worse disease-free survival. At multivariate analysis, only the effect of preoperative hydronephrosis and the use of adjuvant therapy remained to be statistically significant (Table 3).

**Table 3:** Cox regression analysis on factors associated with disease free survival

	Effect size	95% CI		*P*-value
Univariate analysis				
Urinary reconstruction (ileal conduit as reference)	0.661	0.451	0.969	**0.034**
pT stage	3.751	1.189	11.832	**0.024**
pN+	3.513	2.414	5.111	**< 0.001**
Tumur histology: high grade	1.368	0.824	2.27	0.226
Neoadjuvant chemotherapy	1.214	0.826	1.785	0.324
Adjuvant therapy	5.197	3.626	7.448	**< 0.001**
Preoperative hydronephrosis	2.396	1.565	3.669	**0.001**
Preoperative renal impairment	1.461	0.857	2.492	0.164
Diabetes mellitus	0.782	0.492	1.243	0.298
Smoking history	0.999	0.699	1.427	0.995
ASA status	1.365	0.696	2.678	0.791
Age at cystectomy	1.001	0.984	1.019	0.909
Multivariate analysis				
Urinary reconstruction (ileal conduit as reference)	0.861	0.583	1.272	0.453
pT stage	2.447	0.762	7.861	0.133
pN+	1.376	0.894	2.118	0.146
Preoperative hydronephrosis	1.777	1.147	2.754	**0.01**
Adjuvant therapy	2.817	1.844	4.305	**< 0.001**

Significant values are given in bold (*P*-value ≤ 0.05)

*CI* confidence interval, *pT stage* pathology tumor staging, *pN+* pathological nodal positive

**Fig. 2:**
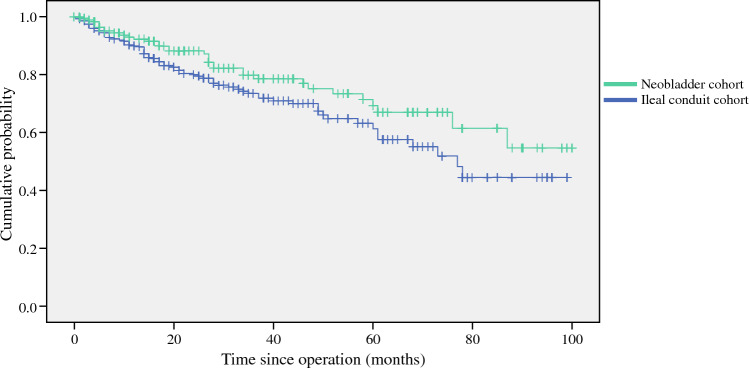
Kaplan–Meier survival curves of overall survival of the ileal conduit and neobladder cohorts. Hazard ratio = 0.670; 95%CI = 0.452–0.993; *p*-value = 0.044. *CI* confidence interval

For overall survival, there was also a similar effect observed, favoring the neobladder group over the ileal conduit group (hazard ratio = 0.670, 95% CI = 0.452–0.993, *p* = 0.044; Fig. 2). Apart from the urinary reconstruction method, univariate analysis identified that tumor T stage, nodal status, tumor high grade histology, need of adjuvant therapy, and the presence of preoperative renal impairment were factors of poor overall survival outcomes. Whereas in multivariate analysis, no factors were identified to be statistically significant (Table 4).

**Table 4:** Cox regression analysis on factors associated with overall survival

	Effect size	95% CI		*P*-Value
Univariate analysis				
Urinary reconstruction (ileal conduit as reference)	0.67	0.452	0.993	**0.046**
pT stage	1.487	1.182	1.872	**0.001**
pN+	2.276	1.468	3.53	**< 0.001**
Tumor histology: high grade	2.487	1.338	4.62	**0.004**
Neoadjuvant chemotherapy	1.294	0.866	1.936	0.209
Adjuvant therapy	2.323	1.58	3.415	**< 0.001**
Preoperative hydronephrosis	1.549	0.949	2.53	0.08
Preoperative renal impairment	1.726	1.006	2.962	**0.048**
Diabetes mellitus	1.272	0.824	1.963	0.277
Smoking history	1.187	0.811	1.738	0.378
ASA status	1.248	0.637	2.446	0.518
Age at cystectomy	1.014	0.994	1.033	0.171
Multivariate analysis				
Urinary reconstruction (ileal conduit as reference)	1.149	0.71	1.86	0.571
pT stage	1.157	0.97	1.38	0.105
pN+	0.688	0.39	1.212	0.196
Adjuvant therapy	0.726	0.424	1.244	0.244
Preoperative renal impairment	1.439	0.781	2.654	0.243
Tumor histology: high grade	1.494	0.757	2.946	0.247

Significant values are given in bold (*P*-value ≤ 0.05)

*CI* confidence interval, *pT stage* pathology tumor staging, *pN+* pathological nodal positive

## Discussion

Historically, urinary diversion with orthotopic neobladder was associated with significant learning curves, with reports of higher rates of significant perioperative complications.^21,22^ This had been one of the key oppressors in pushing for a wider adoption of orthotopic neobladder following RARC. In an analysis involving 2125 patients receiving RARC, the International Robotic Cystectomy Consortium reported an adoption rate of 22% for neobladder use.^23^ In the RAZOR phase III noninferiority trial comparing open versus robotic cystectomy, the adoption rate of neobladder was also 22%.^21^ However, with increasing experiences in RARC, orthotopic neobladder has gained popularity among patients and physicians.^24,25^ Increasing evidence has demonstrated early benefits of RARC. In a 116-patient randomized controlled trial (RCT) comparing RARC versus open radical cystectomy, RARC was demonstrated to be associated with 50% less need of perioperative transfusion.^26,27^ This group of authors also reviewed a set of pentafecta outcomes from the two arms of their RCT^28^ and noted comparable results between open RC and RARC in terms of negative soft tissue margins, lymph node yields, absence of major complications at 90 days and absence of UD-related long-term sequalae, and absence of clinical recurrence. With wider adoption of RARC anticipated, this highlights the pressing need to identify evidence on the optimal method of urinary diversion in RARC.

The current cohort is one of the first that provided data on long-term outcomes of ileal conduit versus orthotopic neobladder in robotic assisted radical cystectomy for nonmetastatic bladder cancer. To the best of our knowledge, there had not been a prospective trial that directly compared these two methods of urinary diversion following RARC. Thus, the results from this multicenter cohort are the closest we can get to identifying the best method of urinary diversion in robotic cystectomy. We identified that the use of orthotopic neobladder was at least comparable to ileal conduit, if not superior, in terms of survival outcomes (DFS and OS). Although operative time in the neobladder group was longer, a larger blood loss or a higher rate of operative complications did not follow. Early outcomes were a tie between the two groups, with neobladder bladder being associated with a lower 30-day readmission rate while the ileal conduit group had a shorter postoperative length of hospital stay. It would be reasonable to comment that the use of orthotopic neobladder in RARC was not associated with inferior outcomes, both short and long term.

Existing literature that compared urinary diversion methods were mainly small volume single center retrospective studies. The main focus of outcomes reported were often functional and quality of life aspects.^29–32^ There was a lack of data on the survival and oncological outcomes. Robotic cystectomy was initially suspected to be associated with worse oncological outcomes compared with open radical cystectomy. This was postulated mainly as a result of higher rate of peritoneal metastasis secondary to pneumoperitoneum,^33^ although this was rebutted in large scale studies.21,34 In RARC followed by orthotopic neobladder, console time was often longer than ileal conduit urinary diversion. There would also be more manipulation of the specimen or bowels during the operation. There would naturally be concerns about whether these factors would translate to increased changes in tumor seeding, hence earlier disease recurrence. Inferences can certainly be made to our finding that the neobladder bladder group was associated with a 14% longer console time than the ileal conduit group. However, this was not related to a worsened oncological outcome in the neobladder group. Comparable DFS and OS between the two groups were identified after adjusting for background confounders, including disease staging.

In a propensity matched population and registry-based retrospective analysis on 5480 patients who received cystectomy (both open and radical cystectomy were included), Su and colleagues reported that orthotopic neobladder was a protective factor for cancer specific and overall survival in the pathological T2 subgroup.^35^ In a retrospective series of 214 radical cystectomies followed by orthotopic neobladder urinary reconstruction (compared with 269 cases of ileal conduit), Yossepowitch and colleagues reported no cancer-specific survival differences between the two groups when pathological stage was adjusted.^36^ In another retrospective series of 768 cystectomy patients,^37^ Stein and colleagues identified the risk of local recurrent tumor to be 5% in neobladder and 9% in cutaneous urinary diversion. It was postulated that urine in contact with urethral urothelium resulted in reduced bacterial carcinogenesis, serving as a protective factor.^38^ All in all, our cohort that especially looked into cystectomy done with the robotic approach added to the conclusion that neobladder use was not associated with worse oncological outcomes.

In our cohort, the neobladder group experienced a higher all-grade complication rates compared with the ileal conduit group. However, the difference was statistically nonsignificant and the rate for serious Clavien–Dindo grade 3–4 complications was similar (16.8% in ileal conduit and 18.0% in neobladder group). A recent multicenter retrospective analysis conducted by the European Association of Urology—Young Academic Urologists: Urothelial Carcinoma Working group involving 555 patients who underwent robotic radical cystectomy^39^ described a similar all-grade complications rate between the two methods of urinary diversion (57% in ileal conduit versus 60% in neobladder). In their analysis, the findings of the neobladder bladder group being associated with longer length of hospital stay and operative time echoed with the finding of the current analysis. However, such operative differences did not seem to translate to negative implications on complication outcomes.

The limitations of the current study should be addressed, with the most obvious limitation being its retrospective nature. Any oncological outcomes observed could be a result of case selection prior to operation. Despite our effort in adjusting the confounding factors (such as age at cystectomy, preoperative renal impairment, and disease staging) via multivariate regression analysis, there could potentially still be unrecognized confounders. Conducting additional statistical maneuvers, such as propensity score matching, could be impractical, with risks of leaving the cohort underpowered to detect recurrence related events. On the other hand, conducting a prospective randomized trial on the choice of urinary diversion would likely be very challenging if not impossible, as this would put a heavy weight on the patients’ quality of life and body image in the long run. Until then, the current analysis would likely remain as a more holistic piece of evidence in this regard. Moreover, the multicenter nature of the current study would bring about heterogeneity as a result of the differences in surgical experience, perioperative care and case selection. However, to mitigate this issue while obtaining a sizeable sample population for analysis via a retrospective study would likely be impractical. Lastly, the current study focused on the long-term survival and oncological outcomes while touching on the short-term perioperative results. There was a lack of quality of life or cost-effective aspects.

## Conclusions

Orthotopic neobladder presented as a robust option for urinary diversion following robotic cystectomy. Short term perioperative and long-term survival outcomes were not inferior to ileal conduit. Prospective studies could be warranted to further investigate its impact.
